# Supplementary material for: Comparative characterization of bronchial and nasal mucus reveals key determinants of influenza A virus inhibition
Source: mSphere. 2025 Aug 25;10(9):e00365-25. doi: 10.1128/msphere.00365-25 (PMC12482186; doi:10.1128/msphere.00365-25)
Supplement: Supplemental material — Supplemental figures and Tables S4 and S5. [file msphere.00365-25-s0001.pdf]

Comparative Characterization of Bronchial and Nasal Mucus Reveals Key Determinants of Influenza A Virus Inhibition

Marie O. Pohl[1], Kalliopi Violaki[2], Lu Liu[1], Elisabeth Gaggioli[1], Irina Glas[1], Josephine von Kempis[1], Carina Messerli[1], Chia-wei Lin[3], Céline Terrettaz[2,4,], Shannon C. David[4], Frank W. Charlton[4], Ghislain Motos[2], Nir Bluvshtein[5], Aline Schaub[,4], Liviana K. Klein[5], Beiping Luo[5], Nicole C. Leemann[6], Markus Ammann[6], Walter Hugentobler[2], Ulrich K. Krieger[5], Thomas Peter[5], Tamar Kohn[4], Athanasios Nenes[2,7], and Silke Stertz[1#]

[1]Institute of Medical Virology, University of Zurich, Zurich, Switzerland

[2]Laboratory of Atmospheric Processes and their Impacts, School of Architecture, School of Architecture, Civil & Environmental Engineering, École Polytechnique Fédérale de Lausanne, Lausanne, Switzerland

[3]Functional Genomics Center Zürich, University of Zurich/ETH Zurich, Zürich, Switzerland

[4]Laboratory of Environmental Virology, School of Architecture, Civil & Environmental Engineering, École Polytechnique Fédérale de Lausanne, Lausanne, Switzerland

[5]Institute for Atmospheric and Climate Science, ETH Zurich, Zurich, Switzerland, Lausanne, Switzerland

[6]PSI Center for Energy and Environmental Sciences, Paul Scherrer Institute, 5232 Villigen, Switzerland

[7]Center for The Study of Air Quality and Climate Change, Institute of Chemical Engineering Sciences, Foundation for Research and Technology Hellas, Patras, Greece

[#] Correspondence to: stertz.silke@virology.uzh.ch

Supplementary Figures and Tables

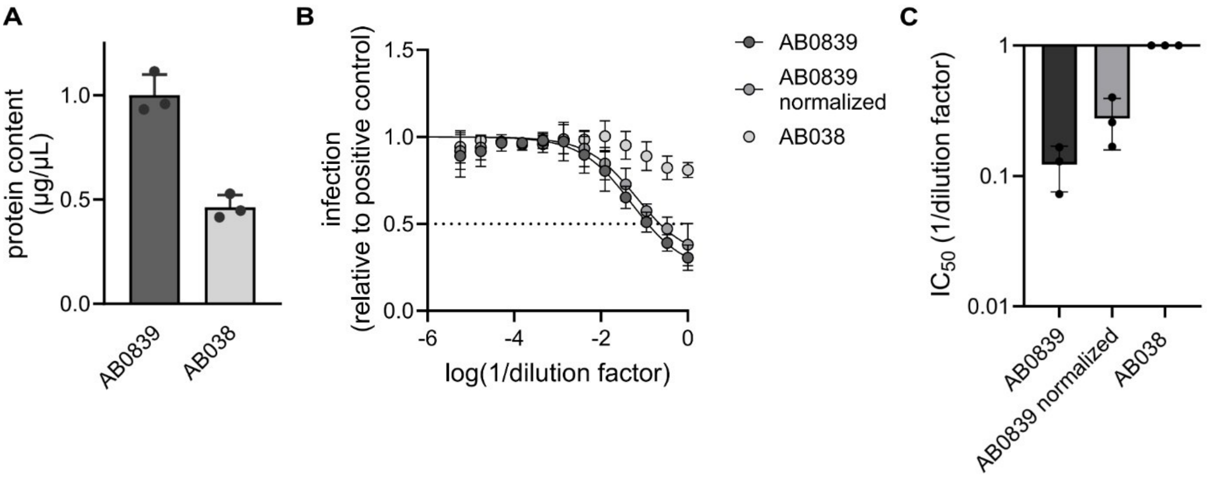

Suppl. Figure 1. A Total protein content of bronchial mucus from AB0839 and nasal mucus from AB038 used in (B) measured by BCA assay. **B** Neutralization capacity of bronchial and nasal mucus against A/Brisbane/59/2007. Bronchial mucus from donor AB0839 was diluted in H2O to match the protein content of AB038 (referred to as AB0839 normalized). IAV was pre-incubated with serial dilutions of mucus for 1 hour on ice. A549 cells were then infected with the virus-matrix mixtures at a MOI of 5. At 7 hours post-infection, cells were fixed and stained for NP. The fluorescent signal relative to the positive control was quantified. Shown are means of three independent experiments. Error bars indicate standard deviation. Non-linear regression was used for curve fitting and to calculate the absolute IC_{50}. **C** Absolute IC_{50} of bronchial and nasal mucus against A/Brisbane/59/2007. For AB038, no absolute IC_{50} could be calculated; values are set to 1.

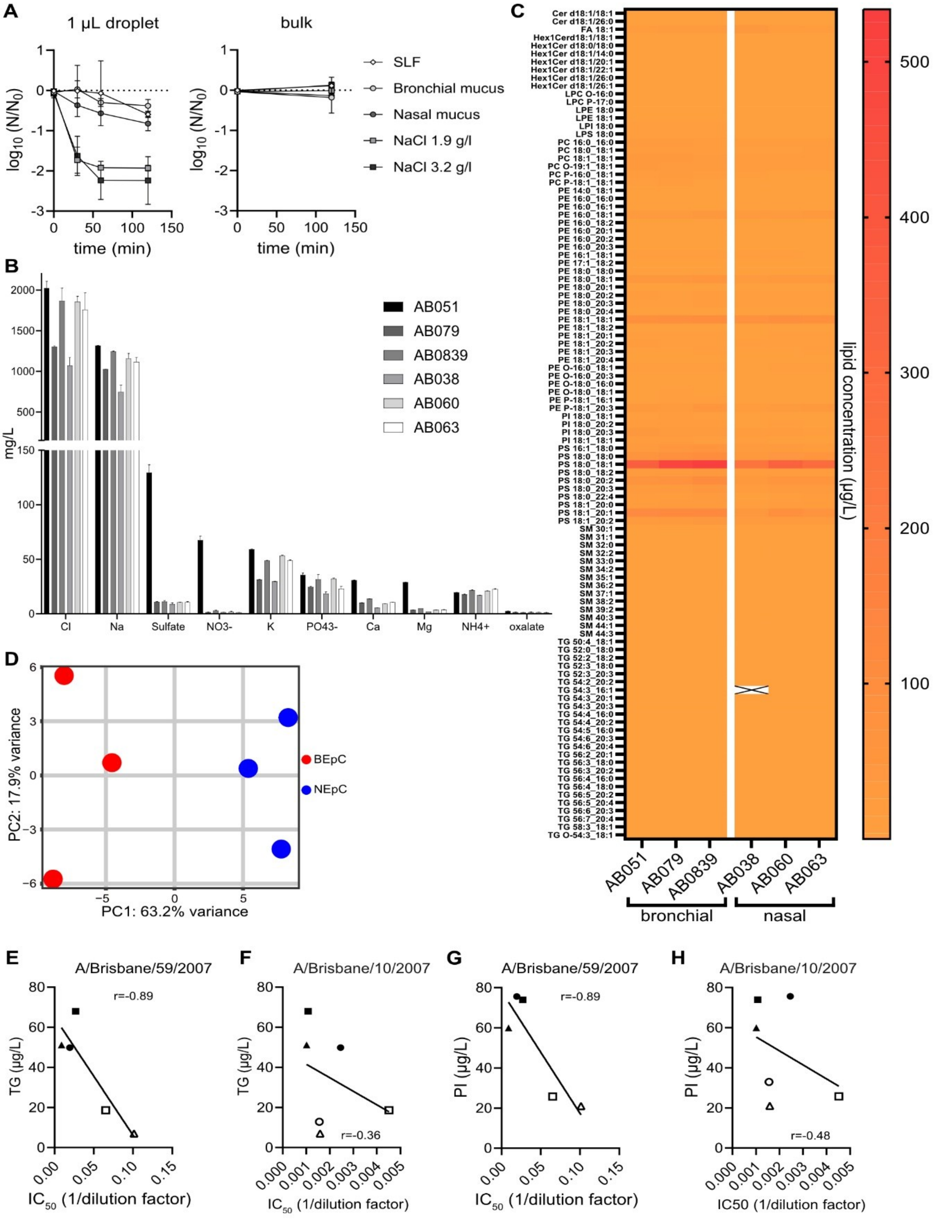

Suppl. Figure 2. A Virus stability in 1-µL droplets. A/Brisbane/59/2007 diluted in saline, SLF, bronchial (AB0839) or nasal (AB038) mucus was incubated for indicated time points at 40% RH and 25°C. Shown is virus decay in droplets and bulk over time relative to 0 min as $\log_{10} N/N_0$. **B** Abundance of different salts in the individual mucus samples. **C** Abundance of lipid

species in bronchial and nasal mucus. **D** PCA of lipid species detected in bronchial and nasal mucus. **E-F** Correlation between TG content and IC_{50} of mucus against A/Brisbane/59/2007 (E) and A/Brisbane/10/2007 (F). **G-H** Correlation between PI content and IC_{50} of mucus against A/Brisbane/59/2007 (G) and A/Brisbane/10/2007 (H). r describes the direction and strength of the correlation. Bronchial mucus samples are depicted as closed symbols (AB051=circle, AB079=square, AB0839=triangle); nasal mucus samples are depicted as open symbols (AB038=circle, AB060=square, AB063=triangle). For AB038, no absolute IC_{50} could be calculated.

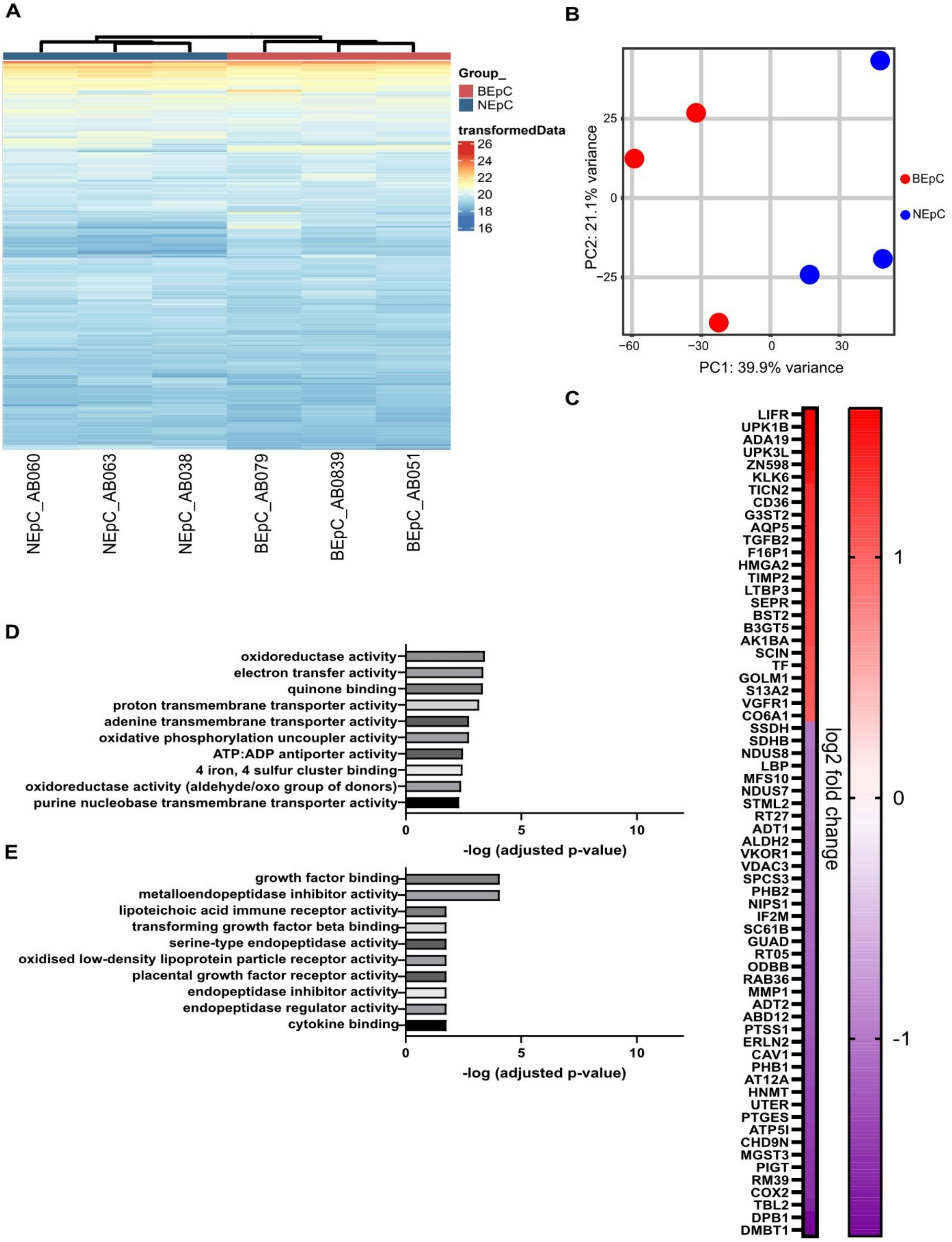

Suppl. Figure 3. A Protein abundance heatmap. Rows indicate proteins; columns indicate mucus samples. Shown is the normalized protein abundance value of the top 1000 abundant proteins. Co-clustering (hierarchical complete linkage, euclidean distance) of samples and proteins was applied. **B** PCA of proteins detected in bronchial and nasal mucus. **C** Heat map

showing the differentially expressed proteins (gene ID) in nasal mucus compared to bronchial mucus. Proteins are ranked based on the log 2-fold change. **D, E** Gene Ontology analysis of the differentially expressed proteins in nasal mucus versus bronchial mucus (C). Analysis was performed with gene profiler and shown are the 10 most enriched GO:MF (molecular function) terms for proteins of lower (D) and higher (E) abundance in nasal mucus.

A

N-glycomics

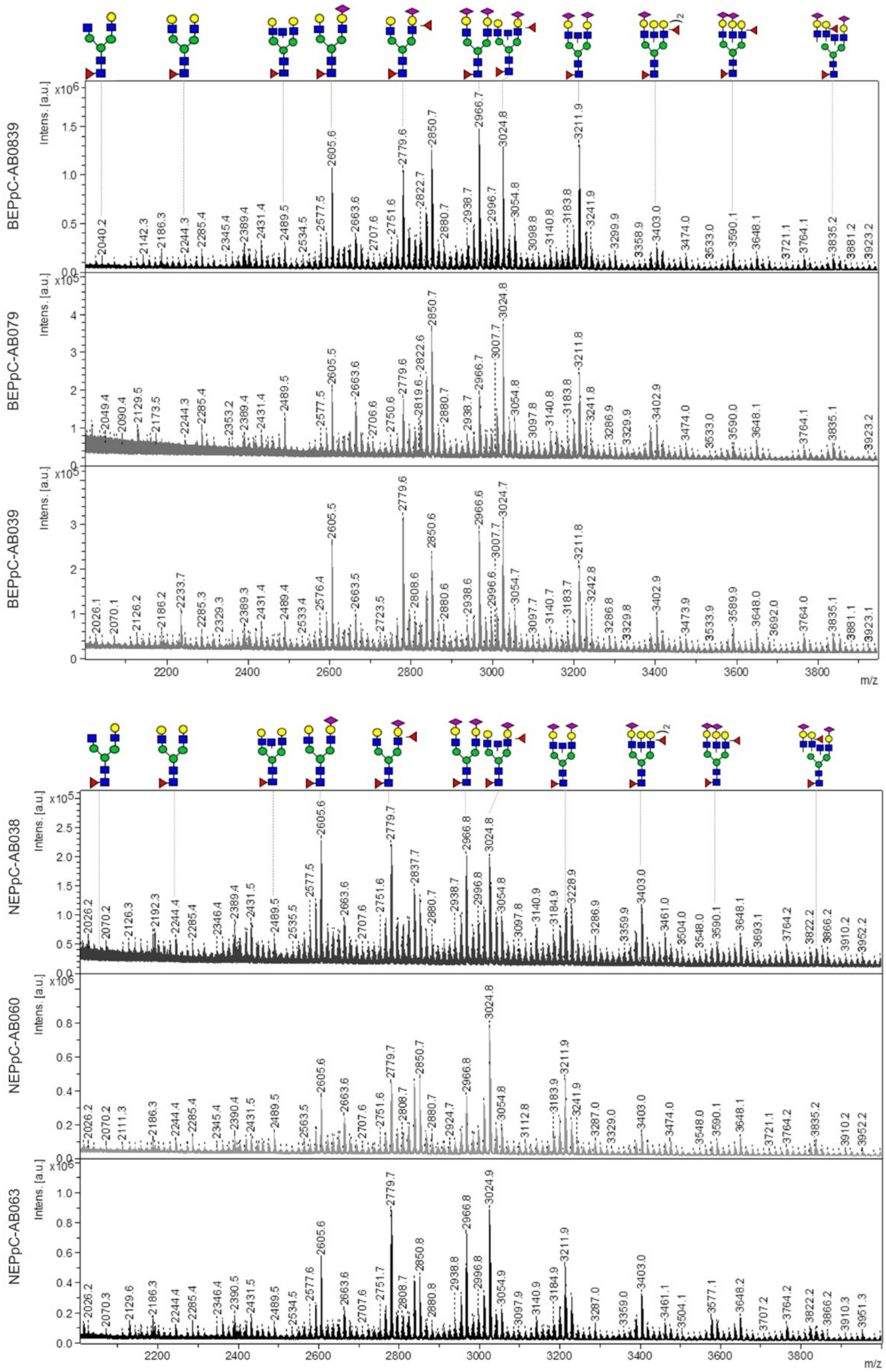

B N-glycomics

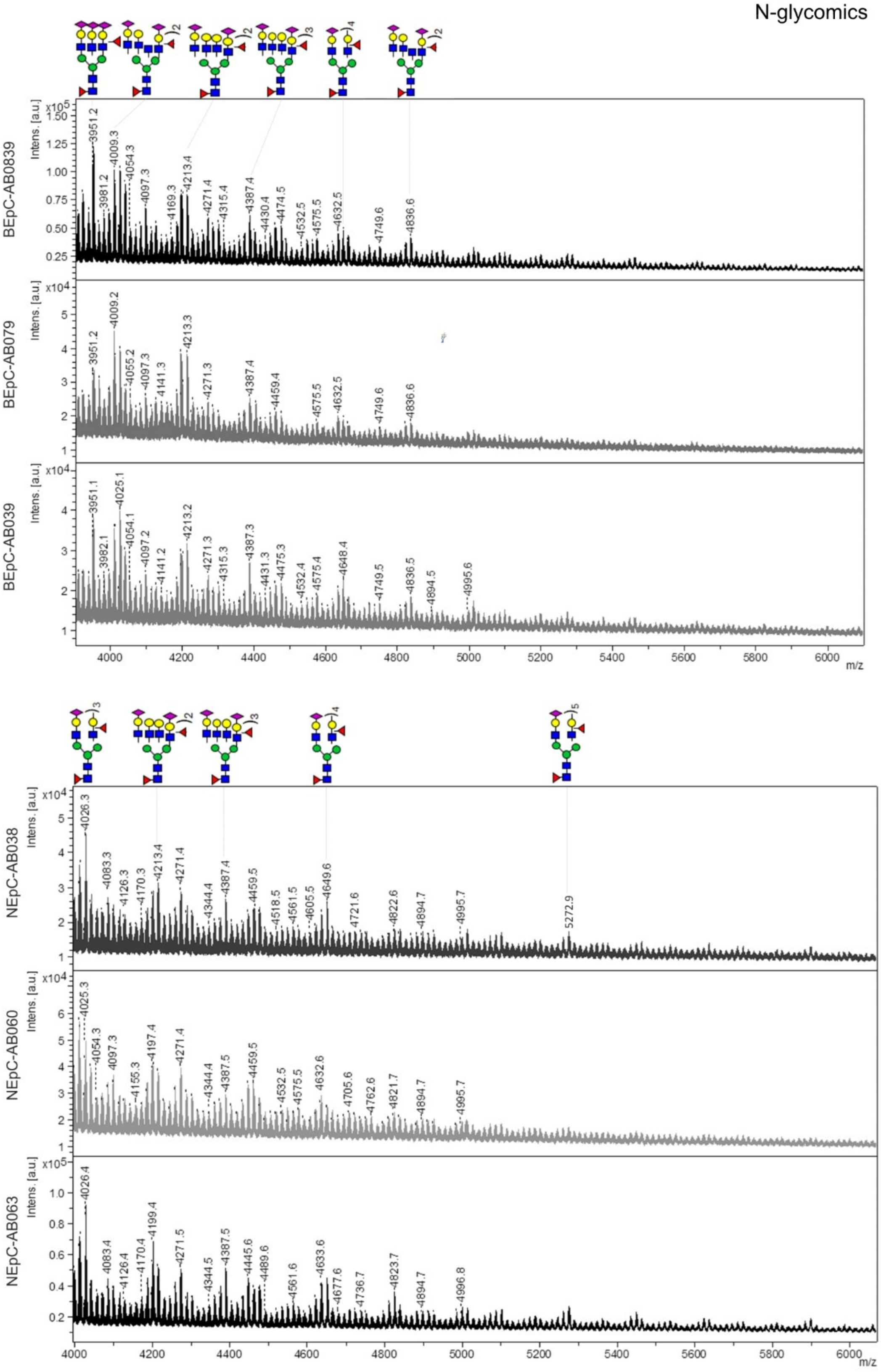

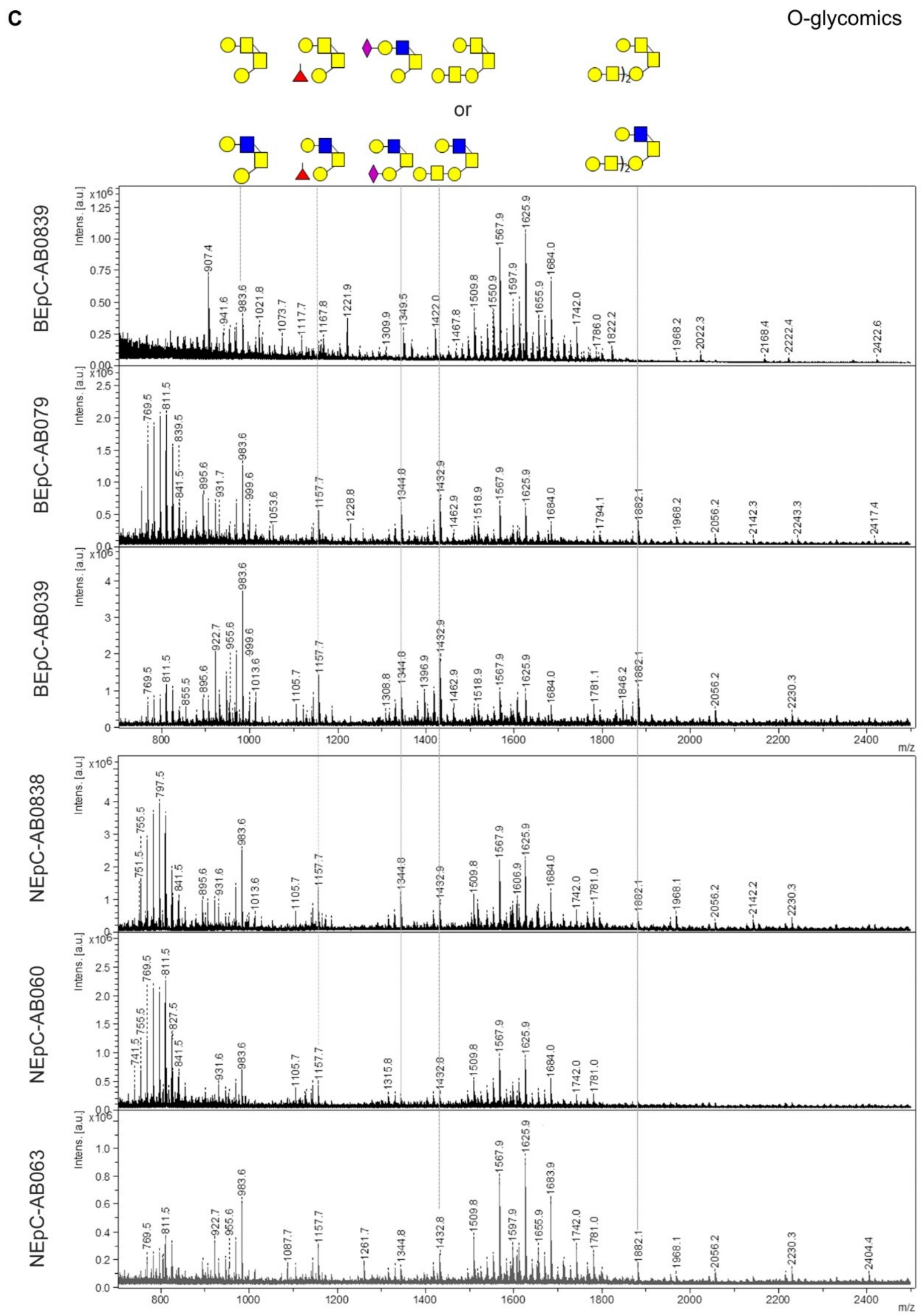

Suppl. Figure 4. A-C Annotated N-glycomic (A, B) and O-glycomic (C) spectra from bronchial and nasal mucus. Top to bottom: BEpC_AB051. BEpC_AB079, BEpC_AB0839, NEpC_AB038, NEpC_AB060, and NEpC_AB063. The annotation is based on the mammalian

glycan biosynthesis pathway. Color codes used to describe the glycans follow the Consortium of Functional Genomics nomenclature: purple diamond: N-acetylneuraminic acid, blue square: GlcNac, green circle: mannose, yellow circle: galactose, red triangle: fucose. $)_x$ indicates the number of the respective structure.

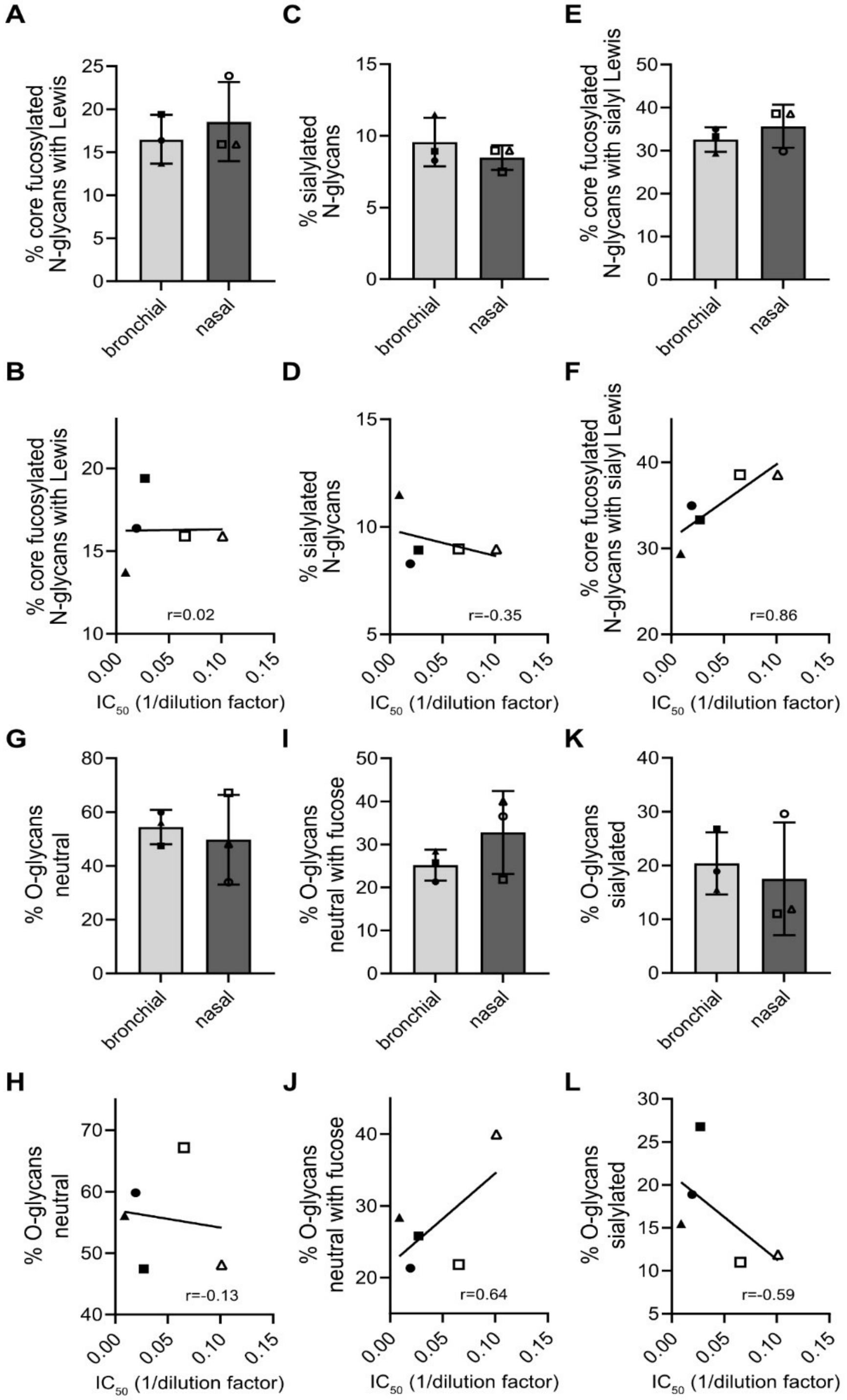

Suppl. Figure 5. A, C, E Abundance of N-glycans depicted as percentage of total N-glycan abundance. Shown are N-glycans with a fucosylated core plus Lewis structure (A), sialylated glycans (B), and fucosylated N-glycans with a sialylated Lewis core (C). **B, D, F** Correlation between N-glycan subtype abundance in mucus (A, C, E) and IC_{50} of mucus against A/Brisbane/59/2007. **G, I, K** Abundance of O-glycans depicted as percentage of total O-glycan abundance. Shown are neutral O-glycans (H), neutral O-glycans with fucose (I), and sialylated

O-glycans (K). **H, J, L** Correlation between O-glycan subtype abundance in mucus (G,I, K) and IC_{50} of mucus against A/Brisbane/59/2007 (Fig 2E). r describes the direction and strength of the correlation. Bronchial mucus samples are depicted as closed symbols (AB051=circle, AB079=square, AB0839=triangle); nasal mucus samples are depicted as open symbols (AB038=circle, AB060=square, AB063=triangle). For AB038, no absolute IC_{50} could be calculated.

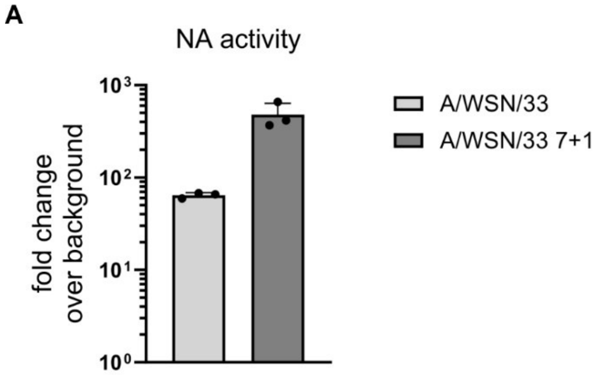

Suppl. Figure 6. A Neuraminidase activity of A/WSN/33 and A/WSN/7+1. Shown is the neuraminidase activity of 10^5 copies of the indicated viruses as fold difference over the background signal from three independent experiments each performed in triplicates.

m/z	Structure		BEpC-AB051	BEpC-AB079	BEpC-AB039	NEpC-AB038	NEpC-AB060	NEpC-AB063
**high mannose**								
1171.669	Hex3HexNAc2	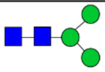		7.07%	3.43%		2.46%	
1579.813	Hex5HexNAc2	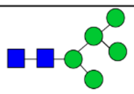	3.84%	3.94%	3.82%	6.96%	5.32%	4.65%
1783.97	Hex6HexNAc2	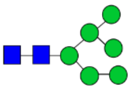	4.74%	3.86%		11.75%	7.93%	7.06%
1988.098	Hex7HexNAc2	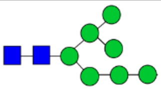	1.15%				1.34%	1.45%
**core-fucosylated complex N-glycans with Lewis structure($HexNAc_nHex_m$+dHex≥2, n≥2, m≥3)**								
1795.122	Hex4HexNAc3dHex1	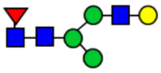				3.80%		
2244.261	Hex5HexNAc4dHex1	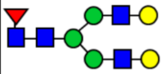	1.03%		0.82%		1.00%	0.81%
2285.29	Hex4HexNAc5dHex1	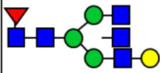	1.08%	1.53%	1.19%		1.24%	0.92%
2418.487	Hex5HexNAc4dHex2	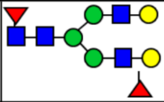					1.10%	
2459.383	Hex4HexNAc5dHex2	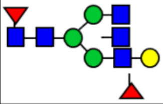	0.95%	1.22%	0.94%		1.05%	0.77%
2489.406	Hex5HexNAc5dHex1	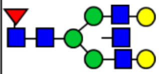	1.45%	2.36%	1.62%	1.28%		0.91%
2663.505	Hex5HexNAc5dHex2	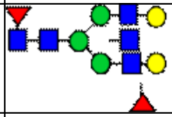	1.86%	3.28%	2.14%	2.10%	2.48%	1.70%
2693.509	Hex6HexNAc5dHex1	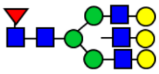	0.96%	1.18%	0.91%	1.11%	0.79%	0.60%
2734.615	Hex5HexNAc6dHex1	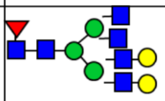						0.50%
2837.67	Hex5HexNAc5dHex3	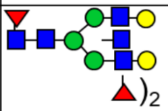		4.98%		3.21%	4.33%	3.13%

2938.634	Hex6HexNAc6dHex1	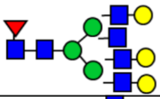	1.17%	1.39%	1.57%	1.48%	1.03%	0.98%
2979.642	Hex5HexNAc7dHex1	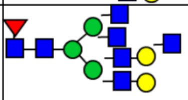	0.80%		0.69%	0.72%	0.55%	0.55%
3112.731	Hex6HexNAc6dHex2	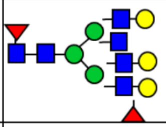	0.91%	1.23%	0.79%	1.18%	0.82%	0.72%
3183.753	Hex6HexNAc7dHex1	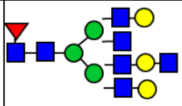	0.91%		1.46%		1.08%	
3215.857	Hex6HexNAc5dHex4	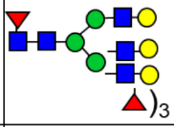		1.59%		1.16%		1.19%
3286.823	Hex6HexNAc6dHex3	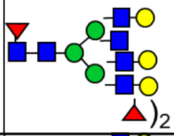	0.84%	1.08%		1.37%	0.95%	0.87%
3387.984	Hex7HexNAc7dHex1	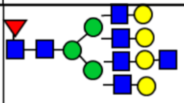				1.51%	0.81%	1.12%
3460.417	Hex6HexNAc6dHex4	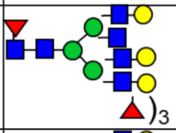	0.54%	0.84%		1.45%		1.00%
3490.988	Hex7HexNAc6dHex3	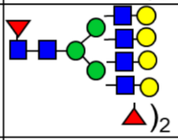		0.76%				
3561.937	Hex7HexNAc7dHex2	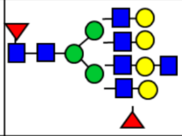	0.65%		0.56%	0.79%	0.51%	0.68%
3736.006	Hex7HexNAc7dHex3	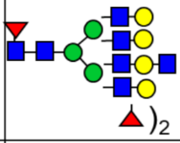	0.53%		0.51%	0.76%	0.43%	0.41%
3807.167	Hex7HexNAc8dHex2	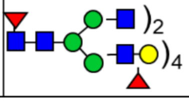			0.52%			

3837.195	Hex8HexNAc8dHex1	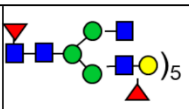				0.91%		
3839.068	Hex7HexNAc6dHex5	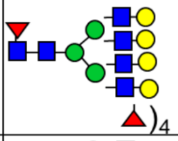	0.50%		0.37%		0.34%	
3910.24	Hex7HexNAc7dHex4	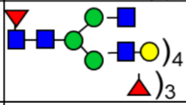				0.68%	0.31%	
3981.248	Hex7HexNAc8dHex3	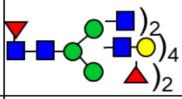				0.57%	0.30%	
4013.322	Hex7HexNAc6dHex6	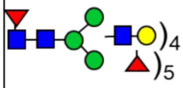					0.33%	
4054.161	Hex6HexNAc7dHex6	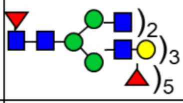	0.51%		0.39%		0.34%	
4155.37	Hex7HexNAc8dHex4	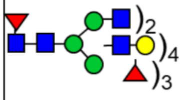					0.30%	
4185.415	Hex8HexNAc8dHex3	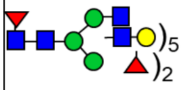						0.43%
4359.429	Hex8HexNAc8dHex4	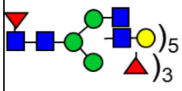				0.56%	0.30%	0.34%
4430.51	Hex8HexNAc9dHex3	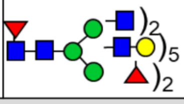						
**complex N-glycan with sialic acid(s) (HexNAc$_n$Hex$_m$+NeuAc≥1,n≥2, m≥3)**								
2186.218	Hex5HexNAc3NeuAc1	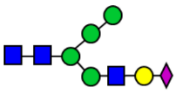	1.07%		1.13%		1.02%	1.10%
2390.38	Hex6HexNAc3NeuAc1	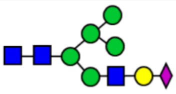		1.35%		1.57%	1.26%	1.31%
2431.364	Hex5HexNAc4NeuAc1	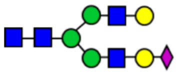	1.52%	1.46%	1.72%	1.89%	1.31%	1.32%
2472.365	Hex4HexNAc5NeuAc1	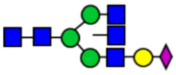	0.73%				0.65%	

2588.431	Hex4HexNAc4NeuAc2	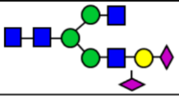	0.76%		0.62%			
2676.505	Hex5HexNAc5NeuAc1	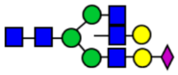	1.20%	1.66%	1.70%	1.22%	1.24%	1.04%
2880.604	Hex6HexNAc5NeuAc1	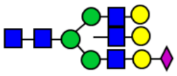	1.15%	1.59%	1.46%	1.26%	1.01%	0.70%
2921.613	Hex5HexNAc6NeuAc1	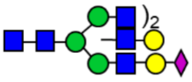	0.64%			0.72%		0.43%
3037.679	Hex5HexNAc5NeuAc2	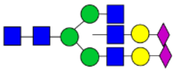	1.00%	1.05%	0.46%		0.82%	0.67%
3153.858	Hex5HexNAc4NeuAc3	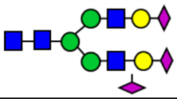				0.69%		
3241.891	Hex6HexNAc5NeuAc2	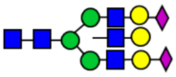			1.39%		0.81%	
3329.825	Hex7HexNAc6NeuAc1	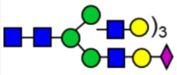	0.61%		0.56%		0.46%	0.43%
3370.952	Hex6HexNAc7NeuAc1	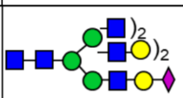			0.39%	0.63%	0.39%	0.42%
4140.369	Hex8HexNAc7NeuAc2	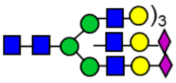					0.29%	
**core-fucosylated complex N-glycanswith sialic acid(s) (HexNAcnHexmdHex1NeuAc≥1,n≥2, m≥3)**								
2156.194	Hex4HexNAc3NeuAc1dHex1	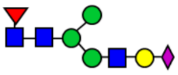	0.94%					0.73%
2360.32	Hex5HexNAc3NeuAc1dHex1	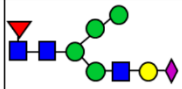	1.09%		1.01%		0.88%	1.22%
2401.348	Hex4HexNAc4NeuAc1dHex1	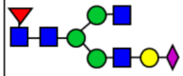	1.02%		1.12%			
2564.485	Hex6HexNAc3NeuAc1dHex1	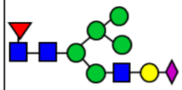		1.00%				
2605.466	Hex5HexNAc4NeuAc1dHex1	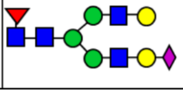	5.19%	4.00%	6.13%	5.14%	3.71%	4.41%

2762.535	Hex4HexNAc4NeuAc2dHex1	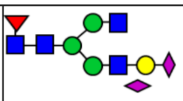	0.86%		0.67%		0.55%	0.53%
2850.599	Hex5HexNAc5NeuAc1dHex1	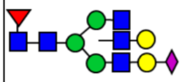	4.81%	7.28%	7.16%	3.00%	4.81%	3.42%
2966.651	Hex5HexNAc4NeuAc2dHex1	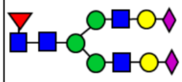	5.71%	3.82%	8.60%	4.75%	3.95%	5.94%
3007.667	Hex4HexNAc5NeuAc2dHex1	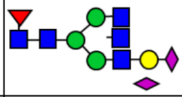	0.91%	0.95%	0.83%	0.83%	0.65%	0.60%
3054.701	Hex6HexNAc5NeuAc1dHex1	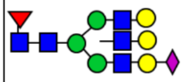	2.21%	2.48%	2.75%	2.26%	1.91%	1.74%
3211.777	Hex5HexNAc5NeuAc2dHex1	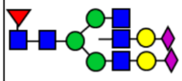	4.21%	5.07%	7.84%	0.76%	4.79%	4.25%
3299.82	Hex6HexNAc6NeuAc1dHex1	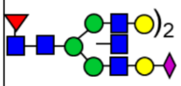	0.85%	1.00%	1.09%		0.73%	0.55%
3415.869	Hex6HexNAc5NeuAc2dHex1	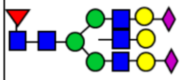	1.02%		1.26%		0.85%	0.94%
3503.914	Hex7HexNAc6NeuAc1dHex1	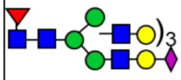	0.67%		0.64%	0.78%	0.41%	0.43%
3777.085	Hex6HexNAc5NeuAc3dHex1	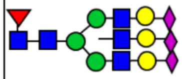	0.50%			0.78%		
3865.073	Hex7HexNAc6NeuAc2dHex1	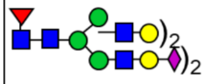	0.58%		0.53%		0.36%	
4198.237	Hex8HexNAc8NeuAc1dHex1	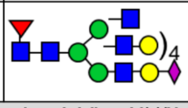	0.63%					
**core-fucosylated complex N-glycans with Lewis and sialic acid(s)(HexNAcnHexmdHex≥2NeuAc≥1, n≥2, m≥3)**								
2779.561	Hex5HexNAc4NeuAc1dHex2	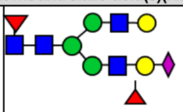	6.36%	3.59%	6.08%	4.93%	4.53%	6.87%
2953.704	Hex5HexNAc4NeuAc1dHex3	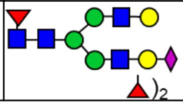		1.54%				2.37%

3024.692	Hex5HexNAc5NeuAc1dHex2	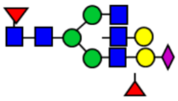	7.00%	7.99%	7.07%		7.93%	7.14%
3140.737	Hex5HexNAc4NeuAc2dHex2	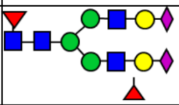	1.33%	1.87%	1.22%	1.80%		1.27%
3198.843	Hex5HexNAc5NeuAc1dHex3	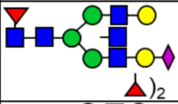		2.38%		1.58%		
3228.784	Hex6HexNAc5NeuAc1dHex2	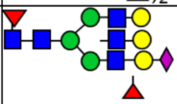	2.15%	1.98%		2.50%	1.98%	2.45%
3269.927	Hex5HexNAc6NeuAc1dHex2	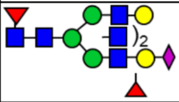				0.66%		
3301.935	Hex5HexNAc4NeuAc1dHex5	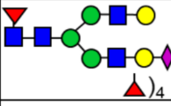				0.75%		
3372.849	Hex5HexNAc5NeuAc1dHex4	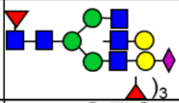	0.68%	0.99%				
3402.874	Hex6HexNAc5NeuAc1dHex3	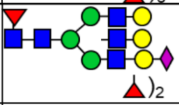	1.76%	2.19%	1.47%	2.54%	1.34%	2.65%
3473.909	Hex6HexNAc6NeuAc1dHex2	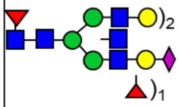	1.02%	1.09%	1.00%		0.97%	0.86%
3577.03	Hex6HexNAc5NeuAc1dHex4	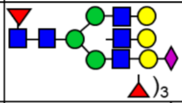		0.85%		0.97%	0.70%	1.32%
3589.958	Hex6HexNAc5NeuAc2dHex2	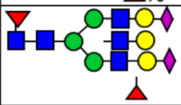	1.45%		1.27%	1.19%	0.99%	1.25%
3647.995	Hex6HexNAc6NeuAc1dHex3	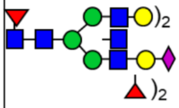	1.23%	1.14%	1.09%	1.60%	1.24%	1.55%
3662.985	Hex5HexNAc4NeuAc2dHex5	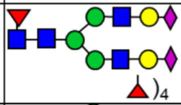	0.69%				0.54%	
3677.995	Hex7HexNAc6NeuAc1dHex2	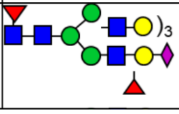	0.80%		0.70%	0.86%	0.55%	0.57%

3402.874	Hex6HexNAc5NeuAc1dHex3	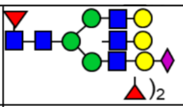	1.76%	2.19%	1.47%	2.54%	1.34%	2.65%
3473.909	Hex6HexNAc6NeuAc1dHex2	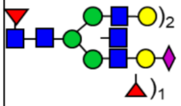	1.02%	1.09%	1.00%		0.97%	0.86%
3577.03	Hex6HexNAc5NeuAc1dHex4	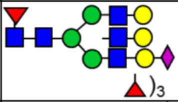		0.85%		0.97%	0.70%	1.32%
3589.958	Hex6HexNAc5NeuAc2dHex2	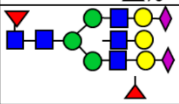	1.45%		1.27%	1.19%	0.99%	1.25%
3647.995	Hex6HexNAc6NeuAc1dHex3	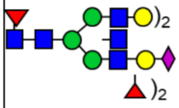	1.23%	1.14%	1.09%	1.60%	1.24%	1.55%
3662.985	Hex5HexNAc4NeuAc2dHex5	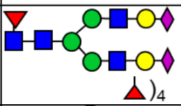	0.69%				0.54%	
3677.995	Hex7HexNAc6NeuAc1dHex2	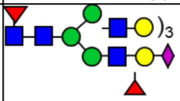	0.80%		0.70%	0.86%	0.55%	0.57%
3996.143	Hex6HexNAc6NeuAc1dHex5	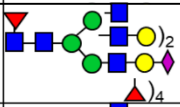	0.54%				0.40%	
4009.151	Hex6HexNAc6NeuAc2dHex3	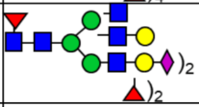	0.80%	1.03%	0.73%		0.61%	
4026.262	Hex7HexNAc6NeuAc1dHex4	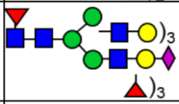		0.90%		1.11%		0.82%
4039.161	Hex7HexNAc6NeuAc2dHex2	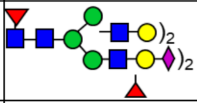	0.69%		0.64%		0.45%	0.44%
4097.197	Hex7HexNAc7NeuAc1dHex3	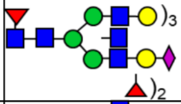	0.55%		0.49%	0.69%	0.42%	0.41%
4170.345	Hex6HexNAc6NeuAc1dHex6	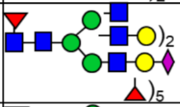				0.58%	0.31%	
4213.24	Hex7HexNAc6NeuAc2dHex3	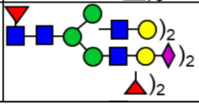	0.72%	0.86%	0.58%	0.84%	0.43%	0.52%

3996.143	Hex6HexNAc6NeuAc1dHex5	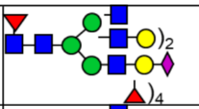	0.54%				0.40%	
4009.151	Hex6HexNAc6NeuAc2dHex3	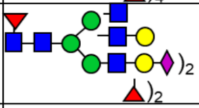	0.80%	1.03%	0.73%		0.61%	
4026.262	Hex7HexNAc6NeuAc1dHex4	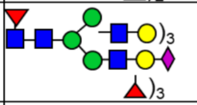		0.90%		1.11%		0.82%
4039.161	Hex7HexNAc6NeuAc2dHex2	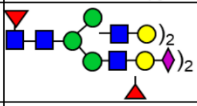	0.69%		0.64%		0.45%	0.44%
4097.197	Hex7HexNAc7NeuAc1dHex3	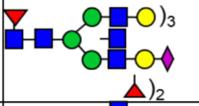	0.55%		0.49%	0.69%	0.42%	0.41%
4170.345	Hex6HexNAc6NeuAc1dHex6	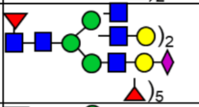				0.58%	0.31%	
4213.24	Hex7HexNAc6NeuAc2dHex3	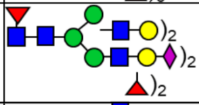	0.72%	0.86%	0.58%	0.84%	0.43%	0.52%
3996.143	Hex6HexNAc6NeuAc1dHex5	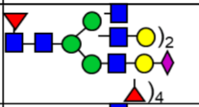	0.54%				0.40%	
4009.151	Hex6HexNAc6NeuAc2dHex3	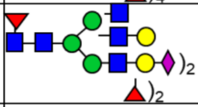	0.80%	1.03%	0.73%		0.61%	
4026.262	Hex7HexNAc6NeuAc1dHex4	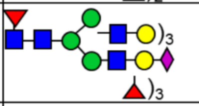		0.90%		1.11%		0.82%
4039.161	Hex7HexNAc6NeuAc2dHex2	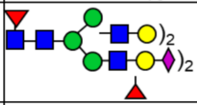	0.69%		0.64%		0.45%	0.44%
4097.197	Hex7HexNAc7NeuAc1dHex3	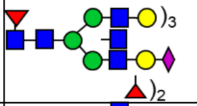	0.55%		0.49%	0.69%	0.42%	0.41%
4170.345	Hex6HexNAc6NeuAc1dHex6	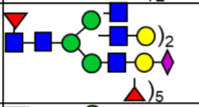				0.58%	0.31%	
4213.24	Hex7HexNAc6NeuAc2dHex3	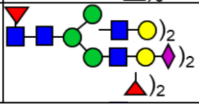	0.72%	0.86%	0.58%	0.84%	0.43%	0.52%

Suppl. Table 4 N-glycan repertoire of bronchial and nasal mucus. The summary of observed mass-to-charge ratio (m/z) and relative abundance (in percent) of N-glycans in samples. The structures were grouped into five: high mannose N-glycans, core-fucosylated complex N-glycans with Lewis structure, complex N-glycan with sialic acid(s), core-fucosylated complex N-glycans with sialic acid(s) and core-fucosylated complex N-glycans with Lewis and sialic acid(s). The principle of grouping is shown in the table. For example, core-fucosylated complex N-glycans with Lewis structure were grouped based on HexNAcnHexm+dHex≥2, n≥2,

m≥3. n was the number of HexNAc and m was the number of Hexose. Since the core structure of N-glycan contains HexNAc2Hex3, n should be more than 2 and m should be more than 3. The annotation is based on the mammalian glycan biosynthesis pathway. Color codes used to describe the glycans follow the Consortium of Functional Genomics nomenclature: purple diamond: N-acetylneuraminic acid, blue square: GlcNac, green circle: mannose, yellow circle: galactose, red triangle: fucose. $)_x$ indicates the number of the respective structure.

m/z	Structure	BEpC-AB051	BEpC-AB079	BEpC-AB039	NEpC-AB038	NEpC-AB060	NEpC-AB063
**netural O-glycans (HexNAc2Hexn)**							
983.636	HexNAc2Hex2	59.82%	23.20%	34.67%	24.01%	32.34%	45.75%
1228.755	HexNAc3Hex2		5.38%				
1432.864	HexNAc3Hex3		18.84%	21.43%	9.88%	15.78%	21.42%
**neutral O-glycans with Fucose(HexNAc2HexnFuc)**							
1157.718	HexNAc2Hex2Fuc	21.30%	10.02%	13.20%	12.28%	24.64%	21.82%
1518.903	HexNAc2Hex2Fuc2		8.73%	5.96%	10.90%		
1606.96	HexNAc3Hex3Fuc		7.05%	9.25%	13.32%	15.35%	
**sialylated O-glycans (HexNAc2HexnNeuAc)**							
1344.806	HexNAc2Hex2NeuAc		14.98%	10.84%	12.94%	11.89%	11.01%
1794.06	HexNAc3Hex3NeuAc		5.97%	4.66%	7.33%		
1968.162	HexNAc3Hex3NeuAcFuc	18.88%	5.83%		9.32%		

Suppl. Table 5 O-glycan repertoire of bronchial and nasal mucus. The summary of observed mass-to-charge ratio (m/z) and relative abundance (in percent) of O-glycans in mucus samples. The structures were grouped into three: neutral O-glycans, neutral O-glycans with fucose, and sialylated O-glycans.
